# Cost-effectiveness of workplace wellness to prevent cardiovascular events among U.S. firefighters

**DOI:** 10.1186/s12872-016-0414-0

**Published:** 2016-11-21

**Authors:** P. Daniel Patterson, Kenneth J. Smith, David Hostler

**Affiliations:** 1 Department of Emergency Medicine, University of Pittsburgh School of Medicine, 3600 Forbes Avenue, Iroquois Bldg., Suite 400A, Pittsburgh, PA 15261 USA; 2 Section of Decision Sciences, Department of Medicine, University of Pittsburgh School of Medicine, Pittsburgh, PA USA; 3 Department of Exercise and Nutrition Sciences, Emergency Responder Human Performance Lab, University at Buffalo, The State University of New York, Buffalo, NY USA

**Keywords:** Cost-Effectiveness, Wellness, Cardiovascular events, Physical fitness

## Abstract

**Background:**

The leading cause of death among firefighters in the United States (U.S.) is cardiovascular events (CVEs) such as sudden cardiac arrest and myocardial infarction. This study compared the cost-effectiveness of three strategies to prevent CVEs among firefighters.

**Methods:**

We used a cost-effectiveness analysis model with published observational and clinical data, and cost quotes for physiologic monitoring devices to determine the cost-effectiveness of three CVE prevention strategies. We adopted the fire department administrator perspective and varied parameter estimates in one-way and two-way sensitivity analyses.

**Results:**

A wellness-fitness program prevented 10% of CVEs, for an event rate of 0.9% at $1440 over 10-years, or an incremental cost-effectiveness ratio of $1.44 million per CVE prevented compared to no program. In one-way sensitivity analyses, monitoring was favored if costs were < $116/year. In two-way sensitivity analyses, monitoring was not favored if cost was ≥ $399/year. A wellness-fitness program was not favored if its preventive relative risk was >0.928.

**Conclusions:**

Wellness-fitness programs may be a cost-effective solution to preventing CVE among firefighters compared to real-time physiologic monitoring or doing nothing.

## Background

The United States (U.S.) fire service is a critical resource for public health and safety. According to the National Fire Protection Association, there are an estimated 30,052 fire departments and 1.1 million firefighters in the U.S. [1]. In 2013, firefighters responded to more than 370,000 residential fires [2]. The occupation is hazardous and poses a unique set of challenges for public health officials and administrators of fire departments. Cardiovascular disease is a leading cause of line-of-duty-death (LODD) among firefighters [3], and from 1995 to 2004, nearly half of all on-duty fatalities were linked to sudden cardiac death [4]. Data show that firefighters experience a disproportionately higher rate of cardiovascular events (CVEs) and mortality than individuals in other occupations, including police and other public safety workers [5]. Although fire suppression is physically strenuous and provides triggers for CVE [6, 7], a high prevalence of overweight, obesity, and physical inactivity among firefighters likely contributes to CVE in fire service [8, 9]. Efforts to reduce CVEs and improve firefighter health have increased; yet there are limited data and guidance on cost-effectiveness of diverse prevention strategies.

There are two commonly discussed approaches to reducing CVEs among firefighters. The first and most widely recognized approach is the adoption of a wellness and fitness program. Since 1997, the International Association of Firefighters, International Association of Fire Chiefs, and National Fallen Firefighters Foundation have advocated mandatory wellness and fitness programs for firefighters. Advocacy stems from an epidemic of overweight and obesity in the fire service – higher than the general population [9]. Where adopted, wellness and fitness programs have generally been successful at improving many metrics of fitness including higher cardiorespiratory capacity and improved body composition [10]. These programs have not been widely implemented in the fire service.

An alternative to wellness/fitness programs is use of real-time physiologic monitoring of firefighters during emergency duties to actively identify firefighters at risk of CVEs. These devices can be worn by firefighters for hours and are capable of collecting basic physiologic parameters such as heart rate, respiration, temperature, and electrocardiogram (ECG). Several devices are commercially available and a large study of physiologic monitoring has been funded by the U.S. Department of Homeland Security (HSHQDC-10-C-00089:P00001). The capabilities of these devices vary but commonly they measure heart rate or ECG and some surrogate of body temperature such as skin temperature or galvanic skin temperature. Devices may have built in alarms to warn the firefighter that they have reached some threshold value of physiology or may transmit data in real time to a monitoring station. Multiple studies have attempted to validate the devices within the context of fire suppression and it seems likely this trend in research of device application and functionality will continue [11, 12].

There is increasing evidence to support mandatory or voluntary wellness and fitness programs for primary prevention of CVE among firefighters and this approach has greater overall benefits for workforce health [13, 14]. However, the incomplete penetration of voluntary or mandatory wellness and fitness programs may require alternate strategies for reducing CVE, such as use of wearable real-time physiologic monitoring technology. There are limited data to support real-time physiologic monitors as a CVE prevention strategy, yet research continues in this area and future devices may support use in the fire service. Administrators at multiple levels of the fire service lack guidance to make informed decisions. What are the advantages and disadvantages of these strategies in the fire service from a cost perspective? We sought to provide information on cost-effectiveness to guide management of CVE risk in the fire service. We used decision analysis to perform a cost-effectiveness analysis comparing a wellness/fitness program, real-time monitoring, and doing nothing.

## Methods

### Study population

Our cost-effectiveness analysis took a fire department/local government administrator perspective, accessing the costs that they are subject to in seeking to prevent firefighter CVEs (Table 1).

**Table 1:** Parameter values used in the model and ranges examined in sensitivity analyses

Parameter	Base Case Value	Range	Reference
Cost			
1-year cost of firefighter CVD-related disability	$325,000	$250,000–$400,000	Ratchford et al., 2014 [18]
Cost of worksite health/wellness/fitness program per employee per year	$144	$130–$150	Baicker et al., 2010 [19], Naydeck et al., 2008 [20]
Physiologic monitoring device			
Initial	~$399–$800/firefighter^%%^	~$399–$800/firefighter^%%^	Quoted values from two commercial vendors^%%^
Yearly thereafter (% of initial cost)	100%	0–100%	Estimate
Cardiovascular event probability (10 years)			
No program	1%	0–10%	cvdrisk.nhlbi.nih.gov, Poston et al., 2011 [9]
Relative risk			
Firefighter fitness program	0.9	0.8–1.0	Estimate
Monitor	0.9	0.8–1.0	Estimate

^%%^Quotes provided to the State University of New York at Buffalo March/April 2015 by Zephyr Technology Corporation and CARRE Technologies

We used a decision model to estimate the cost-effectiveness of options for addressing firefighter CVE risk. Decision analysis compares the value of different decision alternatives [15], and are widely used to address complex questions in diverse industries [16]. From a conceptual standpoint, decision analysis models are a framework for a math problem, with probabilities, costs, and outcomes for each strategy stated quantitatively and the expected value of each strategy calculated, then incrementally compared [17]. To test the robustness of analysis results, sensitivity analyses are then performed, systematically varying model parameters through plausible ranges [17]. When particular components of a decision are unknown, the combination of decision analysis modeling and sensitivity analysis can be particularly useful. In this case, where the benefits and costs of interventions to prevent CVE in firefighters are unclear, our analyses can illustrate which components/parameters related to decisions are most influential in driving analysis results and thus would be the most valuable areas for further research to decrease decision uncertainty. In addition, our analyses of can reveal, through sensitivity analyses, what components of a decision (e.g., probabilities and costs) would need to be in order for a given strategy to be favored. The University of Pittsburgh Institutional Review Board approved our study protocol.

### Measures

**Fig. 1:**
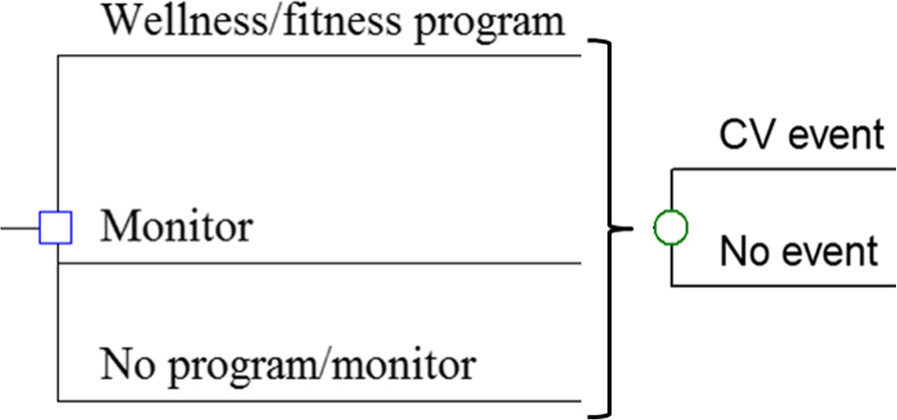
Decision Tree. Figure 1 Notes/Legend: Schematic representation of the decision analysis model. Brackets indicate that the subsequent node will be attached to all prior branches of the decision tree

The effectiveness term of interest was CVE prevented, thus the calculated incremental cost-effectiveness ratio was cost per CVE prevented. We compared two interventions, wellness-fitness programs and real-time physiologic monitoring, which were compared to a third approach where no preventive program was undertaken (Fig. 1). We obtained one-year firefighter cardiovascular disease-related disability costs and cost of a worksite wellness/fitness program per employee per year from the published literature [18–20]. Real-time monitoring device costs were quoted estimates from Zephyr Technology Corporation and CARRE Technologies received March/April 2015. We calculated the 10-year probability of a CVE with the National Heart, Lung, and Blood Institute’s Risk Assessment Tool for Estimating Your 10-Year Risk of Having a Heart Attack (cvdrisk.nhlbi.nih.gov), using the following parameters: Age = 38, Gender = Male, Total Cholesterol = 156 mg/dL, HDL Cholesterol = 38 mg/dL, Smoker = No, Systolic Blood Pressure = 124 mm/Hg, On Medication for HBP = No), obtained from a longitudinal cohort study of firefighters [9]. Based on intervention effectiveness, strategies accounted for potential decreases in CVE rates and were varied in sensitivity analyses (Table 1).

### Statistical analyses

In cost effectiveness analyses, costs and health outcomes are incrementally compared between intervention strategies, using the formula $$ \frac{\mathrm{Cost}\left(\mathrm{x}+1\right)-\mathrm{Cost}\left(\mathrm{x}\right)}{\mathrm{Effect}\left(\mathrm{x}+1\right)-\mathrm{Effect}\left(\mathrm{x}\right)}=\frac{\Delta \mathrm{Cost}}{\Delta \mathrm{Effect}} $$ where Effect = the health outcome of a strategy and x is the rank order of a strategy after ordering by ascending cost [17]. The resulting incremental cost-effectiveness ratios, comparing the wellness-fitness program strategy and the real-time physiologic monitoring strategy, were then compared to firefighter disability costs: strategies with incremental costs per CVE event prevented greater than the disability cost were considered less affordable than programs with lesser costs. We used $2,000,000 as the 10-year disability cost, which was calculated after 3% per year discounting using the low end of the yearly cost range. All parameters were varied individually in 1-way sensitivity analyses over the ranges shown in Table 1 to test the robustness of the base care analysis results, which used the parameter point estimate values. Parameters whose variation caused the favored strategy to change were noted and, of these, selected parameters were examined further in multi-way analyses designed to outline scenarios where strategies could be considered economically reasonable when parameter values were varied over clinically plausible ranges. There is particular uncertainty regarding the effectiveness of wellness programs and physiologic monitors in preventing CVEs among firefighters. Therefore, we performed a series of 2-way sensitivity analyses, simultaneously varying them over their listed ranges in several monitoring cost scenarios.

## Results

In the base case, firefighters with no preventive program have a 1% CVE rate over 10 years. A wellness/fitness program prevented 10% of these, for an event rate of 0.9% at a cost of $1440 over 10 years, or an incremental cost-effectiveness ratio, compared to no program, of $1.44 million per CVE prevented. Physiologic real-time monitors had the same effectiveness as wellness/fitness programs and cost more. In this situation, physiologic monitors are a dominated strategy (the same or lesser effectiveness and higher costs) and would thus not be chosen. The strategy of implementing a wellness/fitness program is favored when using a $2 million per CVE prevented cost-effectiveness threshold.

In 1-way sensitivity analyses, only variation of the CVE probability, the wellness/fitness program CVE relative risk, wellness/fitness program costs, and yearly monitoring costs caused wellness/fitness programs to no longer be favored at a $2 million per CVE threshold. If CVE probability was <0.72% (base case value 1%), wellness/fitness program CVE relative risk was >0.928 (base case 0.9), or yearly wellness/fitness program costs were >$200 (base case $144), no program became the favored strategy. If yearly monitoring costs were <$116 (base case $399), monitoring became the favored strategy.

**Fig. 2:**
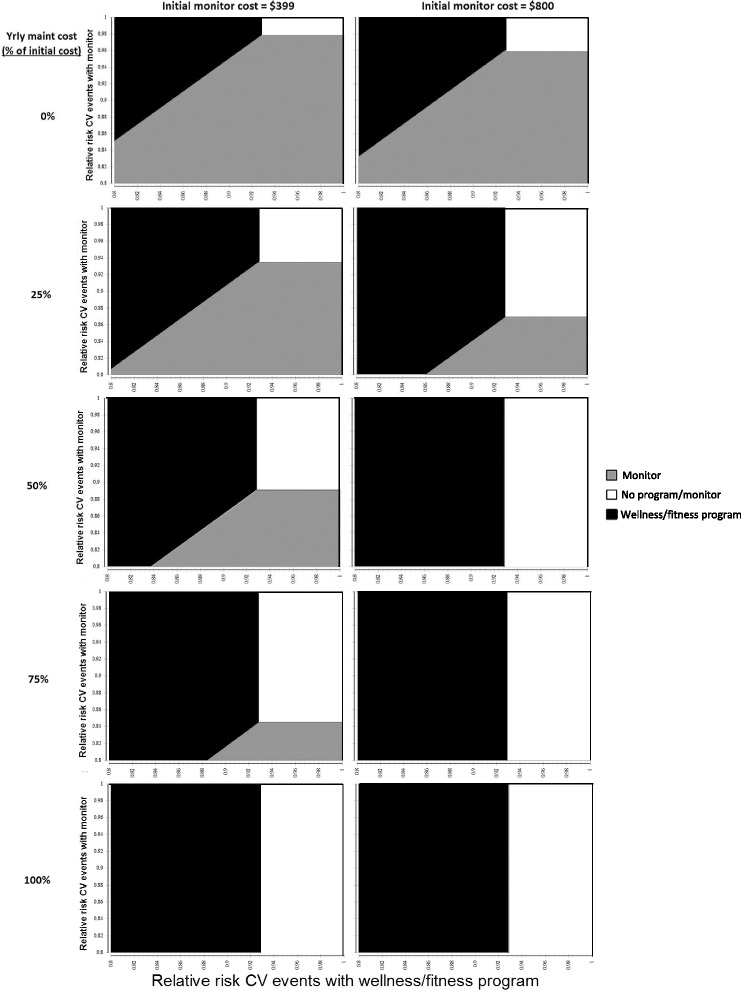
Sensitivity Analysis. Figure 2 Notes/Legend: Sensitivity analysis, varying the relative risk of CVEs with a wellness/fitness program (the x-axis of each graph), the relative risk with a monitor (the y-axis of each graph), the initial monitor cost (columns of graphs), and the yearly monitor maintenance cost as a percentage of the initial cost (rows of graphs). Shaded areas within each graph depict favored strategies when the willingness to pay is $2,000,000 per CVE avoided over a 10-year time horizon

Two-way sensitivity analyses, simultaneously varying the relative effectiveness of wellness/fitness programs and monitoring over a range of monitoring cost scenarios, are shown in Fig. 2. Monitoring was never favored (at $2,000,000/CVE prevented), regardless of its cost, if its effectiveness relative risk is >0.98 or if its yearly maintenance cost is ≥$399. A wellness/fitness program is not favored if its relative risk is >0.928; at these relative risks, the no program strategy is favored if monitoring costs are high.

## Discussion

While increased fitness protects against cardiovascular disease [21], the cost-effectiveness of wellness/fitness programs versus real-time physiologic monitors for CVE prevention on the fire-ground is uncertain. With this in mind, we performed a cost-effectiveness analysis, not to find a definitive answer, but to define what the characteristics of interventions would need to be for them to be considered cost-effective. In our base case analysis, where the relative risk of CVE was 0.9 with either a wellness/fitness program or physiologic monitors compared to no program or monitor (i.e., the status quo in most departments), a wellness/fitness program, but not physiologic monitors, was considered cost-effective when the discounted cost of 10-years of disability, $2,000,000 per CVE prevented, was the cost-effectiveness criterion. This result is sensitive to variation of many model parameters, including intervention-specific CVE prevention and monitoring device cost. Our analysis points out what areas of uncertainty are most important to explore through further research and defines the importance of intervention cost on determinations of cost-effectiveness.

Adoption of wellness/fitness programs within the fire service has been slow but there is increasing awareness of the need to reduce CVE among firefighters. The National Fire Protection Agency – a global non-profit organization that promotes standard development, research, and education for the fire service – maintains a consensus-derived standard (NFPA 1583) that encourages fire departments to establish and provide health-related fitness programs. Firefighters in departments that operate well-developed wellness/fitness programs are healthier, more fit, and have fewer CVE risk factors [13]. Despite support for worksite programs and promising research findings [13], few fire departments require or mandate comprehensive wellness/fitness programs [22]. Our analyses suggest that worksite wellness/fitness programs may be cost-effective for many fire departments and provide the additional benefit of a fitter workforce.

Use of real-time physiologic monitoring is novel and attractive because the greatest risk of CVE among firefighters predominately occurs around emergency activities, such as fire suppression [12]. Firefighters identified to be at risk of a CVE on the fireground may be quickly extricated in hopes of preventing CVE. Use of real-time physiologic monitors may extend beyond the fireground and aid in early identification of distress occurring hours after a high-stress incident. Unfortunately, research exploring use and utility of continuous physiologic monitoring is incomplete and information on cost limited [11, 12]. This creates a dilemma for fire department administrators and physician medical directors. The technology is available and many firefighters do not participate in worksite wellness/fitness programs, thus physiologic monitoring may be considered in spite of limited evidence for effectiveness. We determined that if physiologic monitors have the same effectiveness as wellness/fitness programs and they cost more than fitness/wellness programs, monitors would not be favored. Sensitivity analyses suggest real-time monitors are not favored when monitoring program maintenance costs are ≥$399 per year or if the relative risk of CVE with monitor use is >0.98. With time, the unit costs may decline with increased market penetration and competition, making the monitoring strategy more cost-effective if this occurs.

### Limitations

Our analysis is limited by data availability, which led us to use the analysis to understand and illustrate the importance of uncertain parameter values. We used data published in peer-reviewed manuscripts and quoted values of equipment provided from vendors. We varied all parameters widely in sensitivity analyses. Our findings highlight the need for future research comparing the effectiveness of diverse wellness/fitness programs and understanding whether these programs should be mandatory or voluntary. Although continuous, real-time physiologic monitoring is available and heavily marketed to the fire service, there are no established thresholds of physiology currently measured by these devices (e.g., a critical temperature) that can consistently predict unstable physiology or imminent collapse. In addition to understanding the costs of implementation, future research must elucidate the utility, impact, and cost of real-time physiologic monitoring devices during and post fire-ground events.

In addition, our use of CVE as the effectiveness term will not capture other benefits that might occur as a result of intervention programs, such as improved quality of life, better prevention of, or outcomes from other disease processes, or longer life span. Similarly, our use of a fire department/local government perspective, rather than a broader health care system or societal perspective, might also bias against an intervention.

We did not consider strategies that are uncommonly used in the US, a potential limitation. For this reason we did not consider programs combining wellness efforts and monitoring, wellness/fitness programs that are not on site, or the use of routine periodic cardiac testing in our analysis.

## Conclusions

We conclude that wellness/fitness programs may be a cost-effective solution to preventing CVE among firefighters, being more often favored in the likeliest parameter value ranges. With mature technology, real-time continuous physiologic monitoring may be useful in certain scenarios; however understanding the likelihood of such scenarios will require better and more transparent information on monitoring costs and preventive effectiveness.

## Abbreviations

CVE: Cardiovascular event
ECG: Electrocardiogram
FEMA: Federal emergency management agency
HBP: High blood pressure
HDL: High density lipoprotein
LODD: Line of duty death
U.S.: United States of America
